# Fitness and reduced risk of hypertension—approaching causality

**DOI:** 10.1038/s41371-021-00545-0

**Published:** 2021-05-13

**Authors:** Jari A. Laukkanen, Setor K. Kunutsor

**Affiliations:** 1 Institute of Clinical Medicine, Department of Medicine, University of Eastern Finland, Kuopio, Finland; 2 Central Finland Health Care District, Department of Medicine, Jyväskylä, Finland; 3 Institute of Public Health and Clinical Nutrition, University of Eastern Finland, Kuopio, Finland; 4 National Institute for Health Research Bristol Biomedical Research Centre, University Hospitals Bristol and Weston NHS Foundation Trust and the University of Bristol, Bristol, UK; 5 Translational Health Sciences, Bristol Medical School, University of Bristol, Learning & Research Building (Level 1), Southmead Hospital, Bristol, UK

**Keywords:** Hypertension, Risk factors

Hypertension is one of the leading causes of adverse cardiovascular disease (CVD) outcomes and premature death, which can be prevented by adopting beneficial lifestyle habits, including regular physical activity, maintaining normal body weight, and a healthy diet. Physical activity may prevent the development of hypertension through reduction in body fat mass and weight, improved vascular endothelial function and beneficial modulation of lipid profile, inflammatory markers, and autonomic nervous system balance. It has been recommended that hypertensive individuals should engage in at least 30 min of moderate–intense dynamic aerobic exercise (walking, jogging, cycling, or swimming) for 5–7 days per week in addition to resistance (muscle) training 2–3 days per week, as this kind of exercise program is highly effective in substantially lowering blood pressure [1].

Cardiorespiratory fitness (CRF), a cardiopulmonary exercise testing (CPX) parameter and an index of habitual physical activity, is one of the best measures for assessing aerobic fitness and exercise capacity. Directly measured maximum oxygen uptake (VO2_max_) during CPX is the gold standard for assessing CRF [2]. Cardiorespiratory fitness is an accurate measure of the ability to transport oxygen from the lung to the mitochondria in the muscle level to perform aerobic exercise at the high-intensity level. Left ventricular stroke volume, exercise heart rate, arteriovenous oxygen differences, and muscle metabolism at exercise are major determinants of VO2_max_/CRF levels. Large clinical studies have shown an additional value of CRF assessment in CVD prevention and treatment strategies with good reliability and validity in risk estimation [3]. However, the routine assessment of CRF as a predictor of major fatal and non-fatal CVD outcomes has still remained underused in daily clinical practice; its use involves skills, relatively high costs, and special equipment.

Cardiorespiratory fitness is determined by regular aerobic activity over a long period of time. Possibly more important than aerobic physical activity assessments only, CRF is a separate measure of physical condition and wellness that captures the capacity of the cardiovascular and respiratory systems to supply oxygen to skeletal muscles during incremental exercise intensity to volitional fatigue. Though regular physical activity leads usually to at least some improvement in CRF, genetic and disease-related factors may influence changes in CRF levels and “trainability” between individuals [4]. On the other hand, hypertension development has also a genetic contribution [5], however, we do not have published data showing the possibility that aerobic fitness level and hypertension have a shared genetic contribution. Evidence suggests that approximately half of the variation in CRF is attributed to heritable factors, with the contribution of inherited factors to the response of CRF to physical activity being ~45–50% [3, 4]. The changes in CRF also depend on several other factors such as baseline health and fitness status, type, duration, and intensity of physical activity.

Like moderate and high-intensity physical activity, moderate to high levels of CRF are associated with reduced risk of overall and CVD mortality [6]. Emerging evidence from large-scale epidemiological cohorts also demonstrate that CRF may be associated with future hypertension risk. Given the different definitions of CRF and inconsistent findings by previous studies, the uncertainty regarding a dose–response relationship between CRF and hypertension, and whether obesity could modify the relationship, Cheng et al. conducted a dose–response meta-analysis of all available observational cohorts on the topic [7]. In analysis of 12 studies, the authors showed a 37% risk reduction in hypertension comparing individuals with high versus low CRF, a 15% risk reduction for moderate versus low CRF and an 8% risk reduction for each metabolic-equivalent (MET) increment in CRF. The association remained consistent in several clinically relevant subgroups, though it appeared body mass index (BMI) might be a potential effect modifier of the association.

Only three studies reported on the association between changes in CRF over time and hypertension risk. Comparing participants with an increase in CRF over time to decreased CRF over time, there was a 45% reduction in the risk of hypertension [7]. In our previous studies, we have shown the importance of sustained or improved CRF in contributing to a reduced risk of hypertension and all-cause mortality [8, 9]. Maintaining a good CRF level is one of the most attainable beneficial lifestyle changes that a person can achieve primarily by physical exercise. Due to aging, changes in lifestyle, chronic diseases, and errors in measurements, assessments using baseline measurements of exposure could underestimate the true strength of an association between an exposure and disease outcome in a long-term prospective study due to the phenomenon of regression of dilution bias. Our reproducibility substudies of CRF measurements within the Kuopio Ischemic Heart Disease (KIHD) prospective cohort study showed high within-person variability in CRF levels measured many years apart (regression dilution ratio = 0.58) [10]; which suggests that analyses using only single baseline measurements of CRF will underestimate associations with disease outcomes. This shows that the pooled analysis using baseline values of CRF is likely to be underestimated. The independent association, graded decrease in hypertension risk with increasing CRF levels and the consistency of the associations, suggests causality; however, to demonstrate this requires robust evidence from randomized controlled trials or Mendelian randomization studies.

Obesity (as determined by BMI) and CRF share an inverse relationship and are each associated with cardiovascular outcomes [11]. The authors attempted to investigate if BMI/fatness was an effect modifier of the association between CRF and risk of hypertension by employing meta-regression analysis for studies that adjusted and did not adjust for BMI. Though there was evidence that BMI attenuated the association between CRF and hypertension, the significant association still remained. Given that the current analysis was based on study-level analysis, it is not known whether BMI is just a confounder or an effect modifier. Recent data from the UK Biobank suggested that CRF modified the association between obesity and mortality in men, but this pattern appeared susceptible to biases in women [12]. The same study also showed that low CRF was associated with a twofold higher risk of all-cause mortality in men, irrespective of the level of adiposity [12]. Obese‐fit men were not at an elevated risk of premature mortality compared with normal weight‐fit men and had lower mortality than normal weight‐unfit men [12]. Though there is a wealth of evidence on the relationship between fitness, obesity, and adverse health outcomes, this interplay is still widely debated. However, most of the evidence points to the fact that the favorable effects of lifestyle factors such as physical activity and CRF on adverse outcomes are regardless of the adverse impact of body fatness [13]. Therefore, purposeful weight loss, especially in combination with fitness improvement, may be more beneficial for the risk reduction of hypertension.

The strengths of this study [7] include the novelty, the harmonization of CRF units across studies to maintain consistency and enable pooling and comprehensive analysis. There were several limitations to this meta-analysis, most of which were inherent. Notably, the studies included in the pooled analysis mostly employed indirect methods or nonexercise algorithms for estimating CRF rather than the gold standard measure. There are limitations associated with non-use of the gold standard measure, which include: (i) underestimation and overestimation of CRF at the top and bottom ends of the distribution, respectively, and (ii) a particular equation may not be suitable for all populations [14]. The variation in CRF assessment methods could contribute to biased pooled estimates. Studies did not consistently adjust for the same covariates; hence, whether BMI is a confounder, effect modifier, or mediator of the association is not certain. There was substantial heterogeneity between contributing studies. Furthermore, the current results do not suggest that CRF is a predictor of hypertension because no formal risk prediction analyses were conducted to assess the prognostic relevance of CRF when added on top of risk factors, using measures of discrimination (e.g., Harrell’s C-index) and reclassification (e.g., net-reclassification-improvement). Finally, the findings are observational and do not prove causal relevance.

The findings of Cheng and coworkers are very important and timely as they confirm that hypertension risk can be decreased by maintaining or achieving high CRF levels [7]. High levels of CRF can be achieved through regular physical activity and this should be promoted via population-wide approaches. Based on our recent studies of CPX parameters [6, 15], we would like to inspire the evaluation of novel concepts in this area and era, including the use of the percentage of age-predicted CRF, potentially in the risk assessment of hypertension. Absolute CRF (measured levels without correction for age) is strongly influenced by age and it decreases over the years; hence, its importance as a percentage of the value predicted on the basis of age needs to be considered. In addition to conventional risk factors, the assessment of this parameter may be potentially valuable for hypertension risk evaluation in aging populations. As emphasized by the results of this meta-analysis, the health benefits associated with regular physical activity, which includes aerobic exercise and muscle strength training components, cannot be exaggerated. Efforts to improve CRF with a healthy level of body weight could become a standard part of clinical encounters for the prevention and treatment of hypertension.
